# Comparing the efficacy of morphologic and DNA-based taxonomy in the freshwater gastropod genus *Radix *(Basommatophora, Pulmonata)

**DOI:** 10.1186/1471-2148-6-100

**Published:** 2006-11-23

**Authors:** Markus Pfenninger, Mathilde Cordellier, Bruno Streit

**Affiliations:** 1 Abteilung Ökologie & Evolution, J.W. Goethe-Universität, BioCampus Siesmayerstraße, 60054 Frankfurt/Main, Germany

## Abstract

**Background:**

Reliable taxonomic identification at the species level is the basis for many biological disciplines. In order to distinguish species, it is necessary that taxonomic characters allow for the separation of individuals into recognisable, homogeneous groups that differ from other such groups in a consistent way. We compared here the suitability and efficacy of traditionally used shell morphology and DNA-based methods to distinguish among species of the freshwater snail genus *Radix *(Basommatophora, Pulmonata).

**Results:**

Morphometric analysis showed that shell shape was unsuitable to define homogeneous, recognisable entities, because the variation was continuous. On the other hand, the Molecularly defined Operational Taxonomic Units (MOTU), inferred from mitochondrial COI sequence variation, proved to be congruent with biological species, inferred from geographic distribution patterns, congruence with nuclear markers and crossing experiments. Moreover, it could be shown that the phenotypically plastic shell variation is mostly determined by the environmental conditions experienced.

**Conclusion:**

Contrary to DNA-taxonomy, shell morphology was not suitable for delimiting and recognising species in *Radix*. As the situation encountered here seems to be widespread in invertebrates, we propose DNA-taxonomy as a reliable, comparable, and objective means for species identification in biological research.

## Background

According to Mayr [1], the initial step of any taxonomic work is to "sort that portion of the diversity of the individuals which is encountered into easily recognisable and internally homogeneous groups, and to find constant differences between such groups". In other words, there must be constant, apparent differences between biological entities in order to separate them into natural groups. This is true, no matter whether the taxonomic diagnosis is based on morphological, anatomical, molecular or other traits. In a second step, the so identified groups can be assigned to biological species, either already known to science or not, based on the degree of reproductive isolation to other such groups [2]. Characters that are found to differ constantly among delimited biological species can then be used to re-identify them [1].

Traditionally, morphological traits were used for taxonomy. Recently, however, DNA-taxonomy [3,4] has entered the field, often contradicting traditional views [5]. Therefore, studies comparing traditional taxonomies with DNA-based results are needed [6], because species delimitation and eventual recognition is not only of interest for taxonomists and systematics. Species are the fundamental units in biogeography, ecology, macroevolution, biomonitoring and conservation biology [7-9]. An objective, rigorous taxonomic delimitation of species according to explicit criteria is therefore a necessary prerequisite for many studies in these disciplines. In this contribution, we compared the suitability and efficacy of shell morphology and sequence variation of a mitochondrial gene for taxonomic purposes in a freshwater snail taxon.

The genus *Radix *Montfort 1810, formerly included in *Lymnaea*, is part of the Lymnaeidae family (Basommatophora). It has a Palaearctic distribution, but the results of Remigio indicate a paraphyletic status of the Eurasian taxa on the one side and the mostly East Asian species on the other [10]. The taxonomy and species determination is deemed difficult. Currently, five species, *Radix ampla*, *R. auricularia*, *R. balthica*, *R. labiata *and *R. lagotis *are recognised in North-Western Europe [11]. The most recent taxonomic treatment, summarising previous work, states that species determination based on shell morphology is difficult, unreliable and should be supplemented by anatomical inspections. The latter are, however, also considered unreliable. Indeed, the indicated intraspecific variability of the putatively distinctive anatomical measurements largely overlaps among species [11] and therefore seems to be unsuitable for taxonomic distinction. The issue is further complicated by recent nomenclatorial revisions. The names *R. peregra *and *R. ovata *have lost their validity in favour of *R. labiata *and *R. balthica*, respectively [12], but are still used by some researchers (e.g.[13]).

Species identification in *Radix *is not only of academic interest. The genus is e.g. involved in the transmission of parasitic diseases to humans [14,15]. The study of these diseases is possibly impaired if the specific identity of the snail hosts implicated in larvae transmission cannot be unequivocally determined. Additionally, the presence or absence of certain *Radix *species is used to calculate an indicator of water quality in official assessments [16], which also requires their consistent and correct recognition. This highlights the need for reliable species identifications in this genus.

We compared the suitability of shell morphology and DNA-taxonomy to delimit *Radix *species by focussing on the following issues:

• How many evolutionary lineages of *Radix *exist in North-Western Europe and do they correspond to biological species?

• Does the shell variation of North-Western European *Radix *fall into separate, distinguishable units that correspond to the species descriptions in the taxonomic literature?

• Is shell variation within and among *Radix *lineages species specific or influenced by the environment?

## Results

### MOTU inference with COI

**Figure 1:**
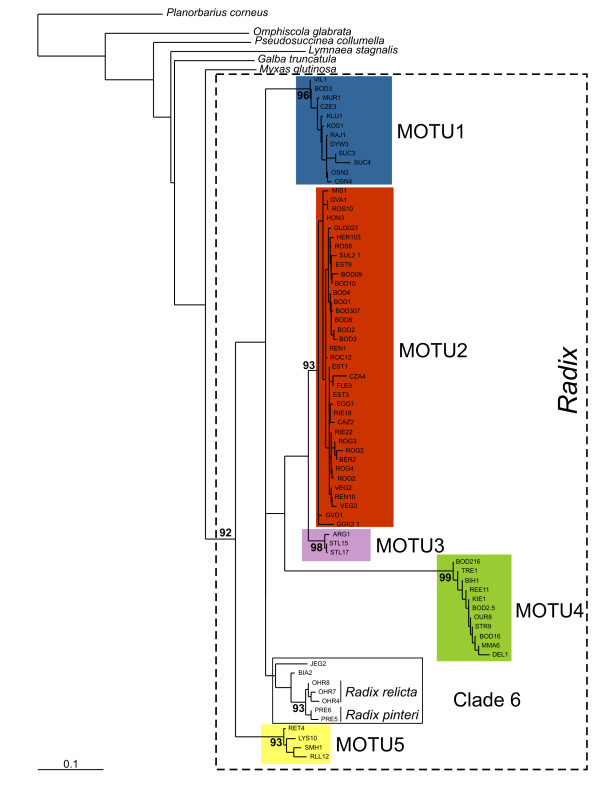
**Neighbour-Joining phenogram of ML-distances among *Radix *COI haplotypes**. MOTU were inferred as least inclusive terminal groups with bootstrap values of 90% or more.

**Figure 2:**
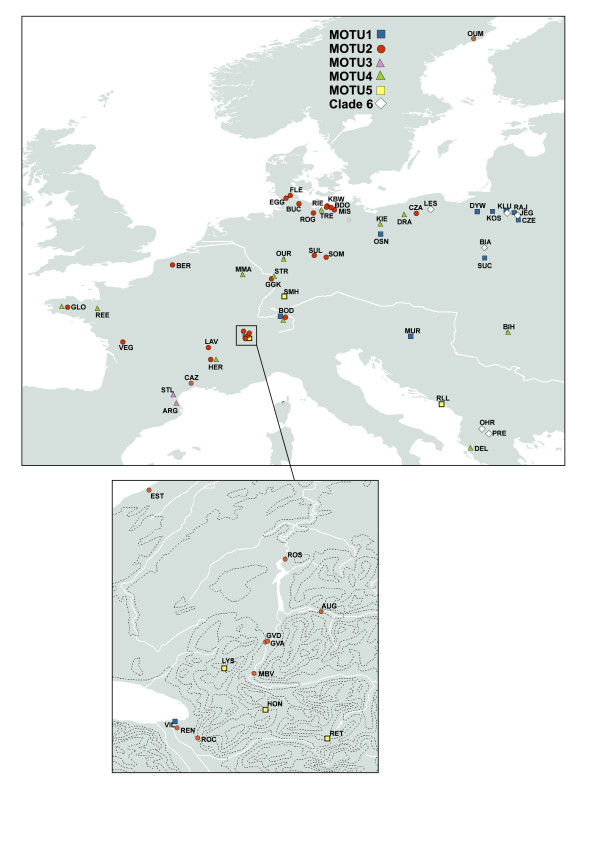
**Geographical distribution of MOTU across the sampling area**. At each sampling site only a single MOTU was found. However, at some locations more than a single site was sampled and yielded different MOTU. The dark lineages in the map-excerpt below are elevation lines.

All 81 *Radix *COI haplotypes formed a monophyletic group relative to the outgroup taxa (Figure 1). According to our definition as least inclusive terminal clades with bootstrap support of 90% or more, we could infer five Molecularly defined Operational Taxonomic Units (MOTU1-5) within *Radix*. They comprised all haplotypes except two from North Poland (Table 1). These two haplotypes grouped with the species *R. relicta and R. pinteri *from Lake Ohrid/Prespa, however, with weak support. For convenience, we refer to the latter group as Clade 6 (Figure 1). The geographic distribution of most MOTU covered the entire range investigated. Only MOTU3 seems to be restricted to the South-West of France and MOTU5 is absent from the Northern parts of the area searched (Figure 2). The average sequence divergence between MOTU ranged from 5% to over 17%, while the sequence diversity within MOTU did not exceed 3% (Table 2). At each sampling site, only haplotypes from a single MOTU were found. For subsequent analyses, we therefore presumed all individuals from the same sampling site to belong to the same MOTU.

**Table 1:** Abbreviations, geographical position, country of origin and MOTU of the 67 *Radix *locations sampled, number of individuals scored for COI, ITS-1 sequence variation and shell shape and whether data on the habitat structure for the site was available.

Abbreviation	Location	Latitude	Longitude	Country	N_COI_	N_ITS-1_	N_morph_	Habitat data	MOTU
OUM	Umea	63.845	20.259	Schweden	4		6		2
FLE	Flensburg	54.699	9.505	Germany	1		7	X	2
EGG	Eggebek	54.614	9.372	Germany	1		-		2
BUC	Groβ Buchwald	54.172	10.073	Germany	1		-		2
KBW	Kühlungsborn	54.149	11.725	Germany	3		12	X	2
RIE	Riedensee	54.149	11.689	Germany	2		24	X	2
BDO	Bad Doberan	54.075	11.936	Germany	2		32	X	2
LES	Lesno	53.945	17.718	Poland	1		8	X	6
MIS	Mistorf	53.884	12.175	Germany	3		4	X	2
TRE	Tressower See	53.851	11.323	Germany	4		4	X	4
KOS	Kosewo	53.834	21.382	Poland	1		3	X	1
DYW	Dywity	53.818	20.477	Poland	2		5	X	1
KLU	Klusy	53.803	22.120	Poland	2		7	X	1
JEG	River Jegrznia	53.730	22.706	Poland	1		-		6
RAJ	Lake Rajgrodzkie	53.730	22.619	Poland	1		7	X	1
ROG	Roggeliner See	53.729	10.940	Germany	3		5	X	2
CZA	Czarne	53.682	16.925	Poland	2		-		2
DRA	Lake Drawsko	53.567	16.22	Poland	1		3	X	4
CZE	Czechowizna	53.333	22.886	Poland	1		7		1
KIE	Kierzkow	52.996	14.811	Poland	1		-		4
OSN1	Osno, River Lenka	52.454	14.871	Poland	3		4	X	1
BIA	Bialobrzegi	51.650	20.962	Poland	2		3	X	6
SOM	Sömmerda	51.236	10.977	Germany	3		-		2
SUL	Sülze	51.087	11.625	Germany	3		49	X	2
SUC	Suchedniow	51.072	20.846	Poland	2		8	X	1
OUR	Oberurff	51.036	9.161	Germany	3		24	X	4
MMA	Maarfelder Maar	50.101	6.758	Germany	5		-		4
STR	Steinrodsee	49.992	8.6	Germany	2		14	X	4
GGK	Groβ Gerau	49.935	8.479	Germany	3		9	X	2
SMH	Stuttgart-Mühlhausen	48.838	9.229	Germany	1		8	X	5
GLO1	Glomel	48.225	-3.404	France	1		8		4
GLO2	Glomel	48.225	-3.404	France	3		14		2
REE	Rennes	48.106	-1.705	France	3		17		4
BOD1	Bodensee	47.709	9.051	Germany	7		-		1
BOD2	Bodensee	47.667	9.213	Germany	11		-		2
BOD3	Bodensee	47.667	9.213	Germany	4		-		4
EST	Estavayer-le-Lac	46.856	6.840	Switzerland	5		20	X	2
BIH	Bihar	46.747	22.210	Romania	1		-		4
ROS	Barrage des Rossens	46.720	7.109	Switzerland	6		24	X	2
AUG	Les Auges	46.615	7.181	Switzerland	6	6	25	X	2
GVD1	Grandvillard	46.555	7.072	Switzerland	4	1	120	X	2
GVD2	Grandvillard	46.554	7.074	Switzerland	3	2	-		2
LYS	Les Lys	46.502	6.989	Switzerland	3	2	13	X	5
MBV	Montbovon	46.492	7.047	Switzerland	3		11	X	2
MUR	Muraszemenye	46.478	16.609	Hungary	1		-		1
HON	Lac de Hongrin	46.419	7.072	Switzerland	3	3	30	X	5
VIL	Villeneuve	46.399	6.890	Switzerland	6	6	19	X	1
REN	Rennaz	46.385	6.895	Switzerland	2	2	27	X	2
ROC	Les Roches	46.364	6.938	Switzerland	2		24	X	2
RET	Lac de Retaud	46.363	7.194	Switzerland	5	5	42	X	5
VEG	River Vegre	46.150	-0.226	France	3		3	X	2
LAV	Laval	45.830	4.804	France	4		38		2
HER1	Herbasse	45.117	4.974	France	2		18		2
HER2	Herbasse	45.117	4.974	France	2		18		4
CAZ	Cazevielle	43.769	3.798	France	2		16		2
STL	St. Laurent de la Cabrierisse	43.070	2.720	France	2		32	X	3
ARG	Argeles sur Mer	42.564	2.902	France	3		2		3
RLL	Rwan Lxjuka à Ljuka	42.541	18.374	Croatia	3		55		5
DEL	Delvinë	39.947	20.091	Albania	1		3		4

**Table 2:** Uncorrected average (± standard deviation) COI sequence diversity (diagonal) and divergence (below diagonal) within and among MOTU.

	MOTU1	MOTU2	MOTU3	MOTU4	MOTU5	Clade 6
MOTU1	0.014 ± 0.004					
MOTU2	0.104 ± 0.014	0.013 ± 0.002				
MOTU3	0.099 ± 0.014	0.050 ± 0.010	0.008 ± 0.004			
MOTU4	0.172 ± 0.019	0.152 ± 0.016	0.152 ± 0.017	0.011 ± 0.003		
MOTU5	0.102 ± 0.013	0.128 ± 0.014	0.129 ± 0.015	0.160 ± 0.016	0.029 ± 0.006	
Clade 6	0.090 ± 0.013	0.098 ± 0.012	0.101 ± 0.013	0.157 ± 0.016	0.122 ± 0.014	0.036 ± 0.006

### ITS-1 variation

**Figure 3:**
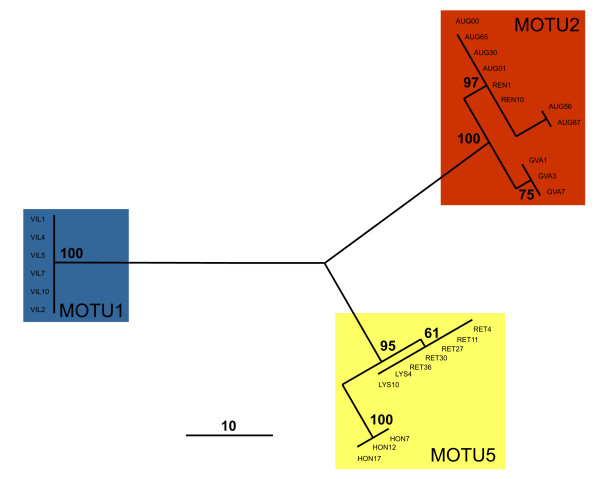
**ITS-1 tree**. Unrooted single most parsimonious tree of nuclear ITS-1 variation from a subset of *Radix *individuals from neighbouring populations in Switzerland (Figure 2). The numbers at the nodes indicate the bootstrap support from 1000 replicates. The three highly supported terminal clades highlighted in gray are congruent to the MOTU as inferred from mitochondrial COI variation (Figure 1).

The ITS-1 sequence variation from the geographically restricted subset sampled in Switzerland revealed three distinct clades with high bootstrap support. These clades were congruent with the MOTU inferred for the respective animals based on COI (Figure 3).

### Crossing experiments

In 4 of the 36 pairings, one or both snails died before reaching sexual maturity. None of the snails kept alone reproduced. MOTU delineation has shown that population GGK and GVD belonged both to MOTU2 and population OUR to MOTU4. All crosses between individuals from the same population yielded viable offspring. All pairings among individuals of the MOTU2 populations (GGK and GVD) were also fertile. However, not a single egg mass was produced in crosses among individuals belonging to different MOTU (OUR snails paired with either GGK or GVD individuals).

### Morphometric analysis

**Figure 4:**
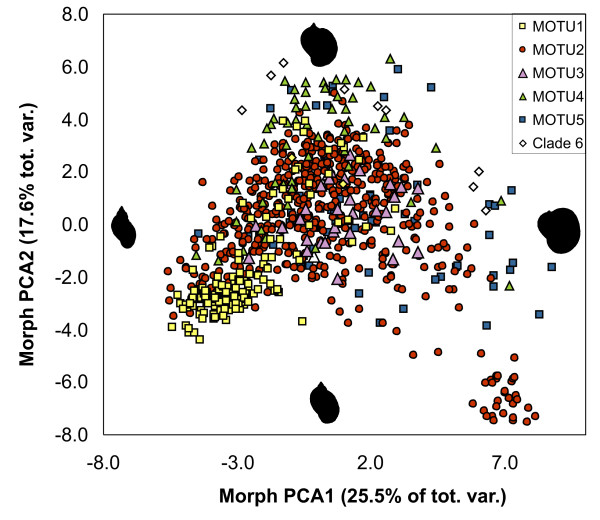
**PCA plot of morphological variation**. Plot of individual scores on the first two Principal Component axes of morphometric shell variation, accounting for 43.1% of the total variation. The affiliation of the individuals to MOTU is indicated, as well as the outline shapes of the most extreme individuals on the respective axes.

The shell shapes of 837 individuals from the field were analysed. PCA extracted two meaningful axes (eigenvalues larger than expected from a broken stick model), representing 25.5% and 17.6% of the total morphometric variance, respectively. The first axis ordinated the shells in a gradient from elongated, slim shape with erect whorls towering over a narrow aperture (negative scores) to a globular shape with barely extruded whorls and a wide aperture larger than the rest of the shell (positive scores). The second axis opposed shells with slightly elevated whorls and wide aperture whose upper rim extends almost in a right angle on the positive side and shells with narrower apertures and immediately descending upper rim on the negative side. Apart from a slightly offset population (KBW) in the lower right quadrant, the morphospace described by these gradients is continuously filled, with most of the specimen positioned in an ellipse from the lower left to the upper right quadrant (Figure 4).

All MOTU occupied a large area of the common morphospace. While MOTU1 and MOTU4 shells were predominantly found to have rather large apertures, the opposite is true for MOTU5. MOTU3 shells figure in the centre of the cloud. MOTU2 individuals were placed everywhere, including the somewhat aberrant KBW population. The morphospace of each MOTU overlaps with all other MOTU (Figure 4). Consequently, a discriminant analysis with MOTU as grouping factor resulted in a poor posterior classification success of less than 50% (details not shown).

### Influence of habitat structure on shell shape

**Figure 5:**
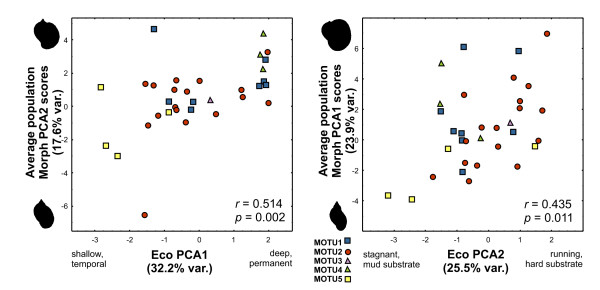
**Covariation of habitat structure with shellshape**. Plot of the population score on habitat structure PCA axes (Eco PCA1 and 2) against a) population average scores on shell morphology PCA axis 2 (Morph PCA2) and b) Morph PCA1.

NPCA on categorical habitat structure variables retained two meaningful axes, summarising 32.2% and 25.5% of total variance. The first axes opposed shallow, temporal water bodies on the negative side and deep, permanent habitats on the positive side. This axis was significantly correlated to the average population scores on Morph PCA2 (r = 0.514, p = 0.002). The second axis delineates a gradient from stagnant waters with muddy substrate to running streams and hard underground. A significant correlation existed between this axis and the average population scores on Morph PCA1 (r = 0.435, p = 0.011). Indicating the MOTU affiliation on the plot of these correlations reveals that MOTU4 occurs preferentially in deep, permanent waters (Figure 5). Otherwise, little structure in the habitat preferences of the MOTU can be detected. This finding is supported by a discriminant analysis with population MOTU as predictor on the habitat structure variables that, albeit being significant, resulted in a poor posterior classification success rate of less than 50% (details not shown).

### Phenotypic plasticity

**Figure 6:**
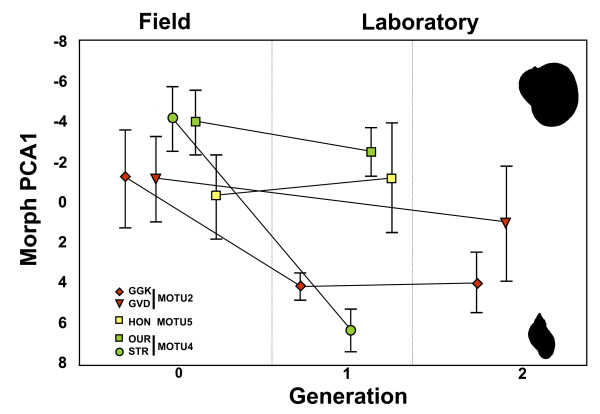
**Shell variation among generations of laboratory bred populations**. Population average scores on shell morphology PCA (Morph PCA1) of five field populations and after one respectively two generations of breeding in the laboratory. The error bars represent 95% confidence intervals.

Rearing populations from three different MOTU (2, 4, and 5) for one or two generations in the laboratory revealed the sensitivity of the developing shell shape to the experienced environmental conditions. In four out of five populations, the average shell shape became narrower, in two cases significantly. One population changed in the opposite direction, but not significantly (Figure 6). Similar, though not significant tendencies toward narrower shells were also observed along the second axes (data not shown).

## Discussion

### MOTU inference and species delimitation in *Radix*

Despite several attempts to characterise *Radix *and other Lymnaeidae species with molecular markers [17-19], this is the first study based on a substantial number of populations and individuals [5]. The monophyly inferred for all presumed *Radix *individuals in this (Figure 1) and other analyses [17,19] suggests that the European species of this genus form a natural taxon. All but two haplotypes were unanimously included in five MOTU according to the definition used. The DNA-taxonomy approach thus succeeded in finding internally homogeneous, recognisable groups of individuals. Following the tree based species delimitation approach of Wiens and Penkrot [6], the lack of apparent gene-flow between populations of different well supported basal lineages strongly indicates the existence of multiple species. The two ungrouped haplotypes from Poland are most similar to those derived from the described species *R. relicta *and *R. pinteri*. Intensified sampling in Eastern Europe would be necessary to determine their mutual relation and their distinctness from the lake Ohrid/Prespa species.

Conservatively assuming an exceptionally fast molecular clock rate, the sequence divergence of 5% among the most similar MOTU2 and 3 suggested that these lineages diverged at least 1 million years ago (Table 2). Such fast rates were suspected for land snails [20], but it is yet unclear whether they apply also for freshwater gastropods. Sequence divergence estimates using more conventional mutation rates of 1.5–2% implied divergence in the Pliocene. Such an ancient divergence among haplotype lineages is usually an indication for longstanding reproductive isolation [21]. The minimum divergence among MOTU exceeded the maximum divergence of 2.9% within MOTU.

Four of the six inferred clades have broad and overlapping geographical ranges (Figure 2). In this regard, *Radix *differs from the freshwater Basommatophora *Ancylus*, in which the inferred cryptic species are confined to specific areas [22]. Given the restricted sampling area and -density, it seems therefore possible that i) some clades like e.g. MOTU3 are actually more widespread and ii) additional clades may exist throughout the distribution range of the genus. Despite often close geographic co-occurrence of different MOTU, we never found two MOTU at the same site. Even though only few individuals were sequenced per site, the joint occurrence of more than one MOTU at a single site seems to be a quite uncommon phenomenon. Differences in micro distribution of two *Radix *lineages in the same mountain lake were also found by Ward *et al*. [23]. This might be due to either different microhabitat preferences, mutual competitive exclusion, rare dispersal events that render multiple colonisations of the same site improbable or – the combination of latter two – monopolisation of the site by the species accidentally arrived first [24].

Shared mitochondrial haplotypes of the same MOTU among often geographically widely separated sampling sites suggested either recurrent gene-flow among populations or their recent common origin [25]. In order to test whether gene-flow among MOTU exists, we have surveyed the nuclear ITS-1 locus on a regional scale in Switzerland, because three of the inferred MOTU (1, 2, and 5) coexist there in close proximity. Nuclear gene-flow, as evidenced by shared haplotypes, seemed to occur only among populations of the same MOTU, but not among different lineages (Figure 3). The reciprocal monophyly and congruence of mitochondrial and nuclear loci despite the possibility to interbreed strongly suggested long lasting reproductive isolation among the lineages. Even though occasional hybridisation can not be categorically excluded based on our data, they seem to be rare events.

These results were corroborated by the crossing experiments among populations of MOTU2 and 4. The complete lack of interbreeding among lineages observed here is consistent with the findings of Wullschleger and Jokela [13]. The lack of offspring produced by snails kept in solitude, does not preclude selfing in the presence of mates or variation among the potential for selfing among different populations [26,27]. Taken all evidence together, the results strongly indicate that the MOTU inferred with COI represent reproductively isolated entities and can therefore be regarded as biological species [2].

### Shell characters are unsuitable to delimit biological entities in *Radix*

The main shell shape gradients extracted from morphometric data correspond to the shell characters used in the taxonomic literature and keys to distinguish among *Radix *species [11,28]. For example, *R. labiata *is presumed to differ from all other *Radix *by a descending upper aperture rim and a slightly inflated last whorl [11]. Such shells can be found in the lower left quadrant of the plot in Figure 4. However, there is a continuous transition between this form and other shell shapes, making it impossible to delimit a morphological entity on the given descriptive criteria. The same is true for all other described species (Figure 4). Therefore, any attempt to find discrete, consistent groups based on shell shape differences is obviously bound to fail, let alone to delimit or identify biological species on the basis of these characters [1,6]. The absence of discrete, homogenous groups based on shell shape showed the unsuitability of these characters for taxonomic purposes in *Radix*. Moreover, the shell variability of the inferred MOTU overlaps to a large extent and is therefore also unsuitable to identify the evolutionary lineages. The failure to find consistent morphological groups and the large mutual overlap in morphospace of evolutionary lineages made it difficult to match the inferred MOTU to described taxonomical entities. One reasonable procedure for such a venture could be to sequence individuals from the type location. There are, however, two major obstacles for this proceeding. First, the type locations are often poorly defined. For example, in the description of *R. auricularia *by Linné in 1758, it is stated that the species "lives in Europe". Given that different species may occur in close proximity (e.g. HER, BOD), even quite precise information may be misleading. Second, the habitat of most *Radix *species is more or less ephemeral. It is therefore questionable whether the present day populations are identical with the described ones several hundred years ago. Consequently, the designation of neotypes, including molecular information and/or tissue deposition appears to be the only feasible strategy to establish the taxonomy of the genus. However, a formal taxonomic revision of the genus was not the aim of the study.

### Influence of the environment on shell shape variation in *Radix*

The environment proved to covary significantly with the shell shape, even though taken rather crudely into account in terms of descriptive habitat structure characteristics. The evolutionary lineage on the other hand had no detectable impact on shell shape, suggesting that the observed variation within *Radix *lineages is little restrained by their phylogenetic history. Because causal relations were not investigated, we cannot say whether the habitat structure has a direct influence on the shell shape or whether covarying factors not taken into account affect the shell development (Figure 5). This raises the suspicion that the reported covariation of differences in the soft body anatomy with shell shape [11] may be also under environmental control and therefore not suited for species delimitation. Further investigations would be necessary to match anatomical differences with species boundaries.

Narrow shells seemed to prevail in temporal, shallow, stagnant waters with soft substrate, while the shells with large apertures were preferentially found in deep, permanent, or running waters on hard surfaces (Figure 5). Large apertures might thus reflect adaptations to predation pressure and/or water current by offering a larger adherence surface for the snail's foot. A phenotypic reaction to particular environmental conditions might also be the reason for the outstanding shell shape of the KBW population, belong otherwise to MOTU2 (Figure 4). The sampling site in a little stream carried an extraordinary load of iron oxide, as evidenced by a typical red precipitate.

The question remained whether the occurrence of similar shell traits in all lineages is due to adaptation to local selection pressures or developmental plasticity in response to environmental conditions (which may be itself under natural selection) common to all *Radix *species. Multiple independent similar adaptations to local selection pressures have been demonstrated for several limnic organisms [29,30]. The fast transition within one generation towards narrower shells under laboratory conditions without water current and predation, however, argues rather for phenotypic plasticity of the shell during growth (Figure 6). A similar change in shell morphology was also observed in a study by Wullschleger and Jokela [31]. Phenotypic response to an unrecorded change in environmental conditions might therefore be a plausible alternative explanation to competitive replacement for the observed transition of narrow shaped shells (identified as *R. peregra*) by broad shaped shells (termed *R. auricularia*) in the field within a single year by Adam and Lewis [32].

## Conclusion

We conclude that the taxonomic distinction of species in the genus *Radix *cannot be based on shell morphology, because the variability is i) continuous, ii) largely overlapping among biological species and iii) phenotypically plastic in response to environmental conditions, as previously suspected [31]. This means that species identifications based on shell morphology have probably not resulted in reliable data. As most species designations of *Radix *in scientific collections, ecological studies or environmental monitoring are based on exactly these characters, results from such studies must be treated with caution. This situation would be embarrassing, but not very serious, if only this particular freshwater gastropod genus would be affected. However, an increasing number of studies e.g. [33-39] have shown that cryptic species or overlapping variability is a quite common phenomenon in invertebrates. Insufficient morphological differentiation among invertebrate species is therefore likely to be taxonomically widespread, resulting in dubious if not outright wrong species identifications and delimitations. In many cases, such misidentifications probably lead to questionable scientific inferences. This situation is likely to be even more severe, since the majority of taxonomic identifications are not made by systematic specialists of the respective taxa, but by researchers interested primarily in other issues [40].

On the other hand, the DNA-taxonomy based on the sequence divergence of short mitochondrial sequences recognised entities that fulfilled the desirable criteria of recognisability and internal homogeneity on the basis of an objective and explicit heuristic [1]. The recognised entities also coincided with biological species, as shown by other lines of evidence. Apart from the manifold practical assets of these approaches, reviewed thoroughly elsewhere [4,41,42], the greatest conceptional advantage of DNA-taxonomy over morphological methods lies in the direct inheritance of the characters used for identification. Unlike many morphological characters, DNA-sequences do not underlie potentially misleading developmental or environmental modifications. Another advantage of this approach is that the evolutionary entities can later be unequivocally re-identified by their COI sequence [43], regardless whether or not the inferred species could be matched to a contemporarily recognised species, belonged to yet undescribed lineages or will be the "victim" of a future taxonomic revision. Moreover, as the DNA sequences and associated digital shell pictures are deposited in Internet based repositories, they are available for further studies, a highly desirable feature of taxonomical data [44,45]. Even an automated MOTU delineation and species re-identification at large scale seems therefore imaginable in the near future [46]. We recognise, however, the danger that also DNA-taxonomy may fail to resolve recently diverged taxa, especially if the species have ancestrally polymorphic mitochondrial haplotypes that do not sort according to subsequent speciation events [47].

The need for reliable species-level identification is contentious [40,48] and DNA-taxonomy and -barcoding could provide it when taxonomic discrimination at this level is warranted. It could also ensure uniform quality of results in studies where the quality of taxonomic data might be compromised by differing taxonomic profusions or opinions among researchers involved. In a way, DNA-taxonomy can make species based research independent of the imponderabilities of present and future taxonomical developments and could keep species based studies comparable over space and time.

## Methods

### Sampling

*Radix *snails were sampled from 60 sites at 57 locations throughout Europe, with emphasis on France, Switzerland, Germany and Poland. At each site, snails were sampled from at maximum 1 m shoreline, the distance between sites at the same location being at least 25 m. Snails were fixed immediately upon sampling in 80% ethanol, and except for those destined for breeding and crossing experiments. The latter were transported in aerated beakers to the laboratory.

### DNA isolation, COI sequencing and MOTU identification

The extracted soft body of the snails was crushed and vortexed in 10% w/v laundry detergent solution for storage at room temperature and tissue digestion [49]. DNA was extracted following the protocol of Winnepenninckx *et al*. [50]. For 169 individuals, a 512 bp segment of the cytochrome oxidase subunit I gene (COI) was amplified with PCR and sequenced. For a subset of individuals (see below), the internal transcribed spacer 1 (ITS-1) from the nuclear ribosomal cluster was additionally amplified and sequenced. An amount of 0.2 to 1 ng total DNA was used as template in polymerase chain reaction (PCR). Specific PCRs were performed with the primers, amplification conditions and temperature profiles shown in [39]. Primers were used for both specific PCR and subsequential automated direct sequencing. PCR products were purified using E.N.Z.A. Cycle Pure Kit (peqlab, Erlangen, Germany) PureLink PCR Purification Kit (Invitrogen, USA). Ten ng per sample were subjected to cycle sequencing using the CEQ DTCS Quick Start Kit (Beckman Coulter, USA). Sequences were analysed on a CEQ 2000 automated DNA Sequencer, Beckman Coulter. In order to verify the results, gene products were sequenced in both directions and the two strands were aligned with SEQUENCE NAVIGATOR 1.0.1 (Perkin-Elmer, Norwalk, CT, USA). Sequences were deposited in GenBank under accession numbers DQ980030–DQ980193. Digital pictures of the shells together with specimen information for most of the sequenced individuals were deposited in the Barcoding of Life Database (MPRAD1-06 – MPRAD139-06). The orthologous DNA sequences were initially aligned using the default settings of CLUSTALW [51] and optimised by eye. The sequences were collapsed to haplotypes prior to phylogenetic analysis. Additionally, COI sequences were obtained from the non-focus species *Radix relicta *(ancient lake Ohrid, Albania) and *R. pinteri *(Lake Prespa, Macedonia), as well as from several other Basommatophora species that served as outgroup. We used the COI data set to infer MOTU, relying on sequence divergence. To this end, the most likely model of sequence evolution and its parameters according to the Akaike information criterion were inferred for the COI dataset using MODELTEST v. 3.6 [52]. The chosen model (GTR+I+Γ) was then used to compute pairwise sequence divergence estimates between all individuals. To visualise the results, an unrooted neighbour-joining (NJ) phenogram was constructed based on the pairwise ML-distance matrix with PAUP 4.10 b [53]. Support of nodes by the data was estimated using the bootstrap [54]. Molecular Defined Operational Taxonomic Units (MOTU) [55,56], were then defined as least inclusive terminal groups with 90% bootstrap support or more, using 1000 bootstrap replicates. This follows the general definition of Operational Taxonomic Units (OTU) as groups of organisms used in a taxonomic study without designation of taxonomic rank. Just as OTU in traditional taxonomy, MOTU do not necessarily equate to biological species, but should be treated as taxonomical hypotheses in need for additional evidence of their mutual reproductive isolation [1].

### Test for congruence among in mitochondrial and nuclear loci

To test for congruence in MOTU inference from mitochondrial and nuclear loci, the sequence variation on the ITS-1 locus of a subset of 27 individuals from neighbouring populations in Switzerland (Table 1) was analysed. These populations were chosen, because three MOTU occurred there in close geographical proximity, allowing potentially gene-flow among these sites. Of the approximately 590 bp fragment amplified, only 217 bp could be unambiguously read, which yielded 47 informative sites. The initial inspection of the sequence alignment revealed that saturation was not an issue, but gaps were likely to be informative. Therefore, parsimony with gaps treated as fifth state was chosen as a means to reconstruct the gene tree, using PAUP 4.10 b [53]. The support of the resulting phylogeny by the data was assessed using the bootstrap [54].

### Crossing experiments

Fifteen to twenty individuals from three populations (GGK, GVD, OUR) were reared until reproduction in the laboratory in different 10 l aquaria in aged tap water at 18–20°C under a 16/8 light/dark regime with food *ad libitum *consisting of boiled lettuce and commercial fish food. Snails from the next generation were isolated before they reached sexual maturity (shell length smaller than 5 mm) and paired with a single other snail in a smaller vessel (1 l) under the same conditions as described above. Pairings were carried out with six replicates for each possible inter- and intra population combination, resulting in a total of 36 attempted crossings. To control for possible self-fertilisation in these hermaphroditic snails, six individuals from each population were raised alone. Only from these three populations, a sufficient number of laboratory reared individuals with known genetic origin was available.

### Morphometric analysis

To assess the shell shape of *Radix *in a repeatable, objective fashion, we used morphometric techniques. Only shells from adult individuals were considered. Shells were placed (with the aperture down) on the glass plate of a scanner and imaged against a black background with a resolution of 300 dpi to greyscale pictures. Resulting images were edited for improved contrast and then transformed to black/white images. The program tpsDIG [57] was used to apply 150 equidistantly spaced points on the shell outline. These points were used to produce a closed outline curve. The shape of the shells was quantified by elliptic Fourier approximation as described by [58]. This technique consists of decomposing a closed contour curve in a two-dimensional plane into a sum of harmonically related sequences. Fourier decompositions are sensitive to location, size and orientation of objects. We consequently used the longitudinal axis of the shells to rotate them into the same orientation. The images were then centred and normalised for size. The decomposition into Fourier series was computed with EFAWin [59], using the algorithms of [60]. The application of 10 harmonics was sufficient to reproduce the outline with high accuracy. As the first three coefficients are trivial, this resulted in 37 Fourier variables. These variables were summarised in a Principal Component Analysis. For correlation analyses with habitat characteristics, the population means of the individual Principal Component scores were computed.

### Assessment of habitat characteristics

To characterise the habitat, the following categorical variables of 35 *Radix *populations (Table 1) visited during sampling were recorded: altitude class (below/above 1000 m), current (stagnant/slow/fast), depth (less/more than 1 m), permanency (ephemeral/permanent), macrophytes (present/absent) and sediment size (mud/sand/hard). We used Nonlinear Principal Component Analysis (NPCA) to summarise habitat score variables of these habitat structure characteristics. NPCA was developed for the analysis of rankable categorical data and can be used in a similar fashion as standard PCA.

### Shell shape variation among laboratory bred generations

Individuals from five populations (GGK, GVD, OUR, HON, STR) were bred separately in the laboratory for one or two generations (two populations reproduced faster) under the conditions mentioned above. Only individuals from these populations could be brought alive in sufficient number into the laboratory. The shells of the deceased adults in each generation were removed from the basin and measured morphometrically as described above. Unfortunately, the shells of the first laboratory generation of GVD were accidentally discarded and could not be analysed.

## Authors' contributions

MP designed the study, sampled a part of the populations, gathered the morphometric data, performed the experiments and analyses and drafted the manuscript. MC sampled also, performed the molecular analyses and was involved in the final preparation of the manuscript. BS provided background information, retrieved relevant literature citations and was involved in the final preparation of the manuscript. All Authors have read and approved the final manuscript version.
